# Analysis of herpesvirus infection and genome single nucleotide polymorphism risk factors in multiple sclerosis, Volga federal district, Russia

**DOI:** 10.3389/fimmu.2022.1010605

**Published:** 2022-11-14

**Authors:** Vera Lezhnyova, Yuriy Davidyuk, Asia Mullakhmetova, Maria Markelova, Alexander Zakharov, Svetlana Khaiboullina, Ekaterina Martynova

**Affiliations:** ^1^ Institute of Fundamental Medicine and Biology, Kazan (Volga Region) Federal University, Kazan, Russia; ^2^ Department of Neurology and Neurosurgery, Samara State Medical University, Samara, Russia

**Keywords:** multiple sclerosis, single nucleotide polymorphism, herpesviruses, ebv, HHV6, risk factor

## Abstract

Multiple sclerosis (MS) is a heterogeneous disease where herpesvirus infection and genetic predisposition are identified as the most consistent risk factors. Serum and blood samples were collected from 151 MS and 70 controls and used to analyze circulating antibodies for, and DNA of, Epstein Barr virus (EBV), human cytomegalovirus (HCMV), human herpes virus 6 (HHV6), and varicella zoster virus (VZV). The frequency of selected single nucleotide polymorphisms (SNPs) in MS and controls were studied. Herpesvirus DNA in blood samples were analyzed using qPCR. Anti-herpesvirus antibodies were detected by ELISA. SNPs were analyzed by the allele-specific PCR. For statistical analysis, Fisher exact test, odds ratio and Kruskall–Wallis test were used; p<0.05 values were considered as significant. We have found an association between circulating anti-HHV6 antibodies and MS diagnosis. We also confirmed higher frequency of A and C alleles in rs2300747 and rs12044852 of *CD58* gene and G allele in rs929230 of *CD6* gene in MS as compared to controls. Fatigue symptom was linked to AC and AA genotype in rs12044852 of CD58 gene. An interesting observation was finding higher frequency of GG genotype in rs12722489 of IL2RA and T allele in rs1535045 of CD40 genes in patient having anti-HHV6 antibodies. A link was found between having anti-VZV antibodies in MS and CC genotype in rs1883832 of CD40 gene.

## Introduction

Multiple sclerosis (MS) is a neurodegenerative disease of the central nervous system (CNS) where the breakdown of integrity of the myelin sheath is believed to be the main cause of nerve tissue damage (1). The disease has chronic progression characterized by the presence of demyelination areas in the brain, called plaques or lesions (2). These plaques are found predominantly in periventricular regions as well as in the temporal and parietal lobes of the brain (3). Destruction of grey and white brain matter was also associated with the disease (4, 5). Clinically, there are several forms of MS: relapsing remitting MS (RRMS), secondary progressing MS (SPMS), primary progressing MS (PPMS), and progressive relapsing (PRMS). The RRMS is characterized by episodes of neurological disability and recovery (4). Clinical symptoms of some RRMS could gradually exacerbate with a steady progression of symptoms, which is referred to as SPMS (6). PPMS is characterized by steady progression of the neurological symptoms without periods of recovery (7, 8).

Pathogenesis of MS is believed to be led by chronic inflammation, which is evident by increased serum mediators of inflammation (9–11). Leukocytes producing pro-inflammatory cytokines can also be found in the brain lesions (12, 13). This inflammation could be triggered by infection leading to activation of glial cells and stimulation of an autoimmune response (14, 15). The patient’s genetics could also contribute to inflammation by reducing control over the inflammatory pathways (16, 17). There are multiple identified risk factors for MS identified however, none of them is selected as a primary cause of the disease. This led to the recognition of MS as a heterogeneous disease where viral and genetic factors were frequently linked to the diagnosis.

The viral etiology of MS was suggested in several studies (18, 19) however, there are multiple viruses suggested to cause MS. Several studies have shown an association between antibodies to the Epstein Barr virus (EBV) and the risk of MS diagnosis (20–22). Further evidence of EBV’s role in MS pathogenesis emerges from finding more frequent disease diagnoses in children born to women with elevated levels of EBV IgG during pregnancy (23). Recently, a large cohort study demonstrated that the risk of MS is 32 folds higher after EBV infection (24). These data provide strong evidence in support of EBV’s role in MS pathogenesis. VZV and human HCMV also suggested contributing to MS risk (25). This suggestion was supported by finding a higher frequency of anti-VZV in MS as compared to controls (26). Interestingly, some studies report the association between anti-HCMV antibodies and reduced risk of MS (27). This protective effect of HCMV infection was explained by modified B cell differentiation and reduced production of pro-inflammatory cytokines in MS (28). Additionally, Human herpesvirus type 6 (HHV-6) was implicated to MS diagnosis (29). It was shown that an increased level of antibodies to HHV-6 directly correlates with a high probability of relapse in RRMS (30, 31). Also, HHV6 RNA was found in periventricular lesions supporting the involvement of HHV6 in MS pathogenesis (32, 33). It appears that there is solid evidence for a pathogenic role of herpesviruses in MS, where viruses could trigger autoimmunity and chronic inflammation (34, 35).

Genetic predisposition is also frequently identified in MS. The role of genetic factors could explain the high prevalence of MS in Northern Europe (36, 37). There are multiple studies suggesting the HLA allele DRB1* 15:01 as the most significant genetic risk factor for MS (38). Other genetic factors contributing to pathogenesis of MS are single nucleotide polymorphisms (SNPs) in genes coding for immune recognition factors, pro-inflammatory cytokines, and cell adhesion molecules. One of them is SNP rs4810485 in the *CD40* gene which is linked to decreased expression of CD40 receptor in antigen presenting cells (APCs) (39). This could prevent ligand-receptor interaction and subsequent inhibition of APCs function (40). Presence of other SNPs, rs2104286 and rs11256593, in the *IL2RA* gene was shown to decrease the expression of the IL2RA receptor on CD4+ T-lymphocytes (41). This could lead to insufficient control over pathogenic CD4+ cells, which contributes to inflammation (41, 42). However, there is still limited data on the role of these and other SNPs on MS diagnosis, and little is known about the combined effect of herpesvirus infection and genetic markers of SNPs on MS diagnosis.

Accumulated clinical and epidemiological data provide evidence for herpesviruses as MS risk factors. The association between MS and herpesvirus infection was shown in Northern Europe (36, 43), Iran (44), the United States (24), Latvia (45) and India (46). MS is diagnosed in the Republic of Tatarstan, a part of the Volga Region of Russia. The prevalence and incidence rates are 36.7 and 5.5 per 100,000 population, which is within the average range in the European part of Russia (47). The role of infection in MS pathogenesis in Tatarstan is limited to a study demonstrating the higher expression of human endogenous retrovirus W in this cohort as compared to that in Great Britain (48). However, our understanding of herpesvirus’s role in MS in Tatarstan and other parts of the Volga Region remains limited. Likewise, our understanding of the association between herpesvirus infection and SNPs linked to MS in this region of Russia is incomplete. To address this knowledge gap, we sought to analyze herpesvirus infection and SNPs alleles in MS and controls from Tatarstan and Samara, two districts within the Volga region. We have found an association between detection of circulating anti-HHV6 antibodies and MS diagnosis. We also confirmed higher frequency of A and C alleles in rs2300747and rs12044852 of *CD58* and G allele in rs929230 of *CD6* gene in MS as compared to controls. We found higher frequency of rs1883832^C^ and rs1535045^T^ in MS female as compared to male in CD40. Fatigue was the only clinical symptom linked to AC and AA genotype in rs12044852 of *CD58* gene. The interesting observation, that supports the role of HHV6 in MS pathogenesis, was finding higher frequency of GG genotype in rs12722489 of *IL2RA* and T allele in rs1535045 of *CD40* genes in patient having anti-HHV6 antibodies. Also, a link between anti-VZV antibodies in MS and CC genotype in rs1883832 of *CD40* gene.

## Materials and methods

### Human subjects

Venous blood and serum samples were collected from MS patients admitted to the Republican Clinical Neurological Center, Republic of Tatarstan, Russian Federation (18/34 male/female; 34.5 ± 10.6 years old) and the Samara State Medical University (SamSMU), the Department of Neurology and Neurosurgery, Samara Region, Russia (38/61 male/female; 38.1 ± 9.14 years old). All 151 MS samples were analyzed (herpesvirus seropositivity status, Expanded Disability Status Scale (EDSS), Multiple Sclerosis Severity Score (MSSS), and presence of fatigue) (**Table 1**). When MS patients were compared to controls, only age matched 124 MS samples were used for analysis (presence of herpesvirus DNA, seroprevalence, and SNPs) (**Table 1**). Age (22/48 male/female; 32.7 ± 13.1 years old) matched control samples from Tatarstan and Samara region were collected. Controls were neurological symptoms and/or MS symptoms free. Patients did not receive immune modified therapy. Demographic and clinical data are summarized in **Table 1**.

**Table 1:** Clinical characteristics of patients and controls included in the study.

	MS total (%)	MS age-matched to controls (%)	Controls
Patients (n)	151	124	70
Male (n)	56	49	22
Female (n)	95	75	48
Age (years)	36.8 ± 9.8	33.7 ± 7.5	32.7 ± 13.1
CIS (n (%))	10 (6.6%)	9 (7.3%)	
RRMS (n (%))	123 (81,5%)	104 (83.9%)	
PPMS (n (%))	4 (2.6%)	3 (2.4%)	
SPMS (n (%))	14 (9.3%)	8 (6.4%)	
Average EDSS	2.87 ± 1.60	2.77 ± 1.57	
Duration of disease (years)	8.82 ± 6.67	8.10 ± 5.76	
Treatment (n)	83	69	
cladribine	12		
rituximab	11		
natalisumab	6		
glatiramer acetate	23		
pegylated IFN-β	31		
No treatment (n)	68	55	
Average MSSS	4.28 ± 2.50	4.20 ± 2.43	
MSSS 0-3 (n (%))	52 (34.4%)	44 (35,5%)	
MSSS 3-6 (n (%))	48 (31.8%)	40 (32.2%)	
MSSS 6-10 (n (%))	40 (26.5%)	29 (23.4%)	
MSSS unknown	11 (7.3%)	11 (8.9%)	

CIS, clinically isolated syndrome, RRMS, relapsing remitting MS; PPMS, primary progressing MS; SPMS, secondary progressing MS; EDSS, Expanded Disability Status Scale; MSSS, Multiple Sclerosis Severity Score; n, number; % - percent from total number of patients. Data is presented as Mean ± SD.

### Ethics statement

This study was done in accordance with the recommendations of Biomedicine Ethic Expert Committee of Republican Clinical Neurological Center, Republic of Tatarstan, Russian Federation (N: 218, 15.11.2012). Signed written informed consent was collected from all subjects. The MS diagnosis was established by the primary physician according to the McDonald criteria (49).

### Enzyme-linked immunosorbent assay

Herpes virus IgG antibodies were analyzed using HHV-6-IgG-ELISA-BEST (cat. D2166, Vector-Best, Russia), VectoVZV-IgG (cat. 2192, Vector-Best, Russia), VectoCMV-IgG (cat. 1554, Vector-Best, Russia), VectoVEB-NA-IgG (cat. 2170, Vector-Best, Russia) ELISA kits according to the instructions. Briefly, two wells of the strip were loaded with 100 μl of a negative control sample, while 100 μl of a positive control sample was added to a separate well. Diluted MS and control serum samples (100 μl; 1:100) were added to the remaining wells in duplicates. The plate was incubated (37 ± 1°C; 30 minutes), washed with 400 μl of washing solution (phosphate buffered saline and 0.5% Tween20 (PBS-T)) 5 times, followed by adding 100 μl of the conjugate. After incubation (37 ± 1°C; 30 minutes), the plate was washed (5x; PBS-T) and incubated with tetramethylbenzidine (TMB) solution (100 μl) for 25 minutes at room temperature. Stop reagent (100 μl) was added to each well before the optical density was measured using an Infinite 200 PRO vertical scanning spectrophotometer (Tecan Trading AG, Switzerland) at the main wavelength of 450 nm and a reference wavelength of 620 nm.

Each test system has a registration number, stating that the product is made specifically for clinical testing and in medical facilities (nevacert.ru). The specificity and sensitivity of each ELISA kit: 100% and 100% for HHV-6-IgG-ELISA-BEST, 100% and 100% VectoVZV-IgG, 100% and 100% for VectoCMV-IgG, 100% and 100% for VectoVEB-NA-IgG, respectively. The registration number is assigned for the product with testing characteristics, such as sensitivity and specificity, similar or superior to currently used tests for detecting anti-herpesvirus antibodies. VectoVEB-NA-IgG kit is marked as Conformité Européenne (CE), indicating that it complies with Harmonized Standards of the European Union (50).

### DNA isolation

Total DNA was isolated using the ExtractDNA Blood & Cells kit (cat. # BC111M, Evrogen, Russia) according to the manufacturer’s manual. Briefly, 100 μl of whole blood was mixed with 100 μl of lysis buffer. GAUSS suspension (10 µl) was added, mixed and incubated at +56°C for 10 min. Then 100 μL of ethyl alcohol (96%) was added followed by mixing with 400 μl of binding solution. Supernatant was separated by centrifugation (1 min at 11000 g) and transferred on the column membrane. The membrane was washed by Washing buffer and DNA was eluted by Elution buffer and collected by centrifugation. Qualitative and quantitative analysis of the isolated DNA was done using Denovix DS-11+Spectrophotometer (DeNovix Inc., USA).

### Quantitative polymerase chain reaction analysis

Herpes virus DNA analysis was done using RealBest DNA HHV-6 (cat. D-2150, Vector-Best, Russia) RealBest DNA VZV (cat. D-2185 Vector-Best, Russia) RealBest CMV DNA (cat. D-1598, Vector-Best, Russia) RealBest EBV DNA (cat. D-2198, Vector-Best, Russia) kits according to the manufacturer’s instructions. PCR-RT was carried out using Real-time CFX96 Touch (Bio-Rad, USA). For each reaction, 30 ng of total DNA was used. The PCR conditions were as follows: 50°C for 2 min, 95°C for 2 min, followed by 50 cycles of 94°C for 10 sec and 60°C for 20 sec. Fluorescence was measured at 60°C using Real-time CFX96 Touch (Bio-Rad, USA). The FAM detection channel was set to register the process of DNA amplification of the internal control sample and the ROX detection channel was set to detect EBV, VZV, HCMV and HHV-6 DNA.

### Genotyping analysis

A total of 16 SNPs were selected in 16 genes: rs113516541, rs11575594 and rs11575584 (*CCL27* gene), rs12722489 and rs2104286 (*IL2RA* gene), rs6897932 (*IL7RA* gene), rs12044852 and rs2300747 (*CD58* gene), rs929230 (*CD6* gene), rs9282860 (*STK11* gene), rs12708716 and rs2041670 (*CLEC16A* gene), rs763361 (*CD226* gene), and rs1883832 and rs1535045 (*CD40* gene). The selection of genes and SNPs was based on data indicating their potential association with risk for MS (42, 51–57). SNPs analysis was done by allele-specific PCR (AS-PCR). Primers were synthesized by Evrogen (Russia). For AS-PCR, a “ScreenMix” reaction mix (Evrogen, Russia) and 20 ng of genomic DNA were used. The AS-PCR parameters were optimized for each set of SNPs primers. Typical AS-PCR results are presented in **Supplemental Figure 1**. The reaction was carried out using a C1000 Touch 96 amplifier (Bio-Rad, USA). Separation of AS-PCR products was done by gel electrophoresis. AS-PCR products were detected in the GelDoc XR+ gel-documenting system (Bio-Rad, USA).

### Statistical analysis

Analysis of the genotype and allele frequencies in MS and control groups was done using the Fisher’s exact test. The strength of associations was assessed using the odds ratio (OR, (lower 95% confidence interval; upper 95% confidence interval). The qPCR and ELISA data analysis was carried out using the two-tailed Fisher’s exact test. Аanalysis of MSSS and EDSS was done using the Kruskal-Wallis test. p<0.05 values were considered as significant.

## Results

### MS and controls

One hundred fifty-one MS cases (56 males and 95 females; average age 36.8 ± 9.8) were included in this study. MS diagnosis was established according to the 2010 Revised MacDonald’s Diagnostic Criteria for MS (58). There were 123 RRMS cases, 4 cases with PPMS, 14 patients with SPMS (**Table 1**), and 10 patients diagnosed with CIS. The mean duration of disease was 8.82 ± 6.67 years. Expanded Disability Status Scale Score (EDSS) and Multiple Sclerosis Severity Score (MSSS) were 2.87 ± 1.60 and 4.28 ± 2.50, respectively. Additionally, 83 patients received disease modifying therapy (cladribine, natalisumab, glatiramer acetate, and pegylated IFN-β) while 68 patients had no treatment.

### Herpesvirus antibody and DNA in blood of MS and controls

To demonstrate the prevalence of herpesvirus infection we selected age matched 124 MS and 70 control, serum antibodies for HCMV, HHV6, EBV, and VZV were analyzed (**Table 2**). We found that antibodies to HCMV, HHV6, EBV, and VZV were in 84.7, 63.7, 97.6 and 87.9% in MS patients, respectively. In controls, circulating antibodies for HCMV, HHV6, EBV, and VZV viruses were in 82.9, 45.7, 92.9, 80.0%, respectively. There was a higher prevalence of only anti-HHV6 antibodies in MS as compared to controls (OR=0.48 (0.26; 0.87), p = 0.02). There was no difference in MS and controls having antibodies to HCMV, EBV, and VZV. Next, we analyzed the herpesvirus seroprevalence in two groups of MS: age-matched (124 samples) and not matched (27 samples) with controls (Supplemental **Table 1**). The control age-matched MS was 33.7 ± 7.5 years old, while the other group was older, 51.5 ± 4.6 years old. We found no difference in seroprevalence between these two groups. These data suggest that the herpesvirus infection has a limited contribution to MS diagnosis in older age.

**Table 2:** Analysis of anti-herpesvirus antibody and DNA in MS and controls.

		MS n (%)		Control n (%)		Fisher’s exact Test
Method	Herpesvirus	+	-	+	-	p value
ELISA	HCMV	105 (84.68%)	19 (15.32%)	58 (82.86%)	12 (17.14%)	0.84
	HHV6	79 (63.71%)	45 (36.29%)	32 (45.71%)	38 (54.29%)	**0.02**
	EBV	121 (97.58%)	3 (2.42%)	65 (92.86%)	5 (7.14%)	0.14
	VZV	109 (87.90%)	15 (12.10%)	56 (80.00%)	14 (20.00%)	0.15
qPCR	HCMV	0 (0.00%)	124 (100.00%)	0 (0.00%)	59 (100.00%)	1
	HHV6	1 (0.81%)	123 (99.19%)	1 (1.69%)	58 (98.31%)	0.54
	EBV	1 (0.81%)	123 (99.19%)	1 (1.69%)	58 (98.31%)	0.54
	VZV	29 (23.39%)	95 (76.61%)	13 (22.03%)	46 (77.97%)	1

ELISA, enzyme-linked immunosorbent assay; qPCR,ndash; quantitative polymerase chain reaction; HCMV, human cytomegalovirus; HHV6, human herpes virus 6; EBV, Epstein-Barr virus; VZV, varicella zoster virus; n- number of patients; % -percent from total number of patients.

Herpesvirus DNA was analyzed in mononuclear leukocytes (**Table 2**) from 124 MS and 70 age matched controls. HCMV, HHV6, EBV, and VZV DNA was found in 0, 0.81, 0.81 and 23.4% MS, respectively. Interestingly, HCMV, HHV6, EBV, and VZV DNA was also demonstrated in 0, 1.7, 1.7 and 22.0% controls. We found no difference in herpesvirus DNA detection between MS and controls (**Table 2**).

Additional analysis revealed a lack of differences in herpesvirus DNA detection in MS and controls when groups were separated based on EDSS, MSSS, duration of the disease and treatment status (**Supplemental Tables 2, 3**). The frequency of anti-herpesvirus antibody detection did not differ in MS patients based on EDSS, MSSS and disease duration (**Supplemental Table 2**). Similarly, there was no association between the detection of herpesvirus DNA and MS patients’ treatment status (**Supplementary Table 3**).

Analysis of sex of MS and controls revealed a lack of difference in sero-prevalence to HCMV, HHV6, EBV, and VZV (**Supplemental Tables 4A, B**). To analyze the link between age and HHV6 antibody prevalence, patients and controls were divided into young (≤35 years old) and old (>35 years old) groups. We have found higher frequency of anti-HHV6 antibodies in older MS patients as compared to the same age controls (65.0% vs. 40.7%; p=0.04). We also found higher frequency of VZV DNA detection in MS patients as compared to controls in both age groups. We conducted an additional analysis using 45 years old cut-off to determine whether older age would be more associated with differences in herpesvirus prevalence. A significantly higher frequency of anti-HHV6 antibodies was found only in young (≤45 years old) as compared to the same age control. There was no difference in the detection of VZV DNA between MS and control in both age groups. These data indicate that VZV could play a role in younger MS.

### SNPs analysis in MS and controls

**Figure 1:**
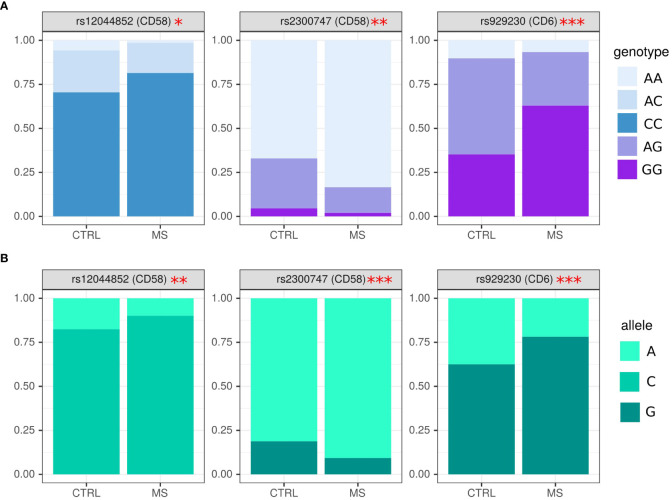
Analysis of genotype and allele frequency in rs2300747, rs929230 and rs12044852 SNPs in MS and controls. **(A)** – genotypes, **(B)** – alleles. SNPs analysis was done by AS-PCR. Data is presented as proportion of representation of genotype/allele. There were 151 MS and 70 controls samples used for the analysis. The analysis was done using Fisher’s exact test. *** - p<0.005, ** - p<0.05, * - p<0.1.

In this analysis, we have used 124 MS samples and 70 age and sex matched controls. Genetic markers agreed to Hardy–Weinberg equilibrium proportions in the control population (p>0.05). Several SNPs were selected for analysis in MS and controls. We found a higher frequency of CC in rs12044852 and AA in rs2300747 genotypes in CD58, while genotype AC and AG in respective SNPs were more often found in controls (**Figure 1A**) (OR=2.92 (0.54; 24.51), p=0.05 and OR=0.41 (0.21; 0.80), p=0.01). AG genotype in rs929230 of CD6 gene was more frequent in controls, while in MS, GG was found more often (OR=0.86 (0.31; 2.37), p=0.0002).

The frequency of alleles concurred with data on genotype where rs2300747^A^ and rs12044852^C^ in CD58 (OR=0.44 (0.26; 0.76), p=0.005, OR=1.94 (1.12; 3.34), p=0.02, respectively), and rs929230^G^ in CD6 gene (OR=2.14 (1.42; 3.23), p=0.0003) were more often found in MS as compared to controls (**Figure 1B**).

**Figure 2:**
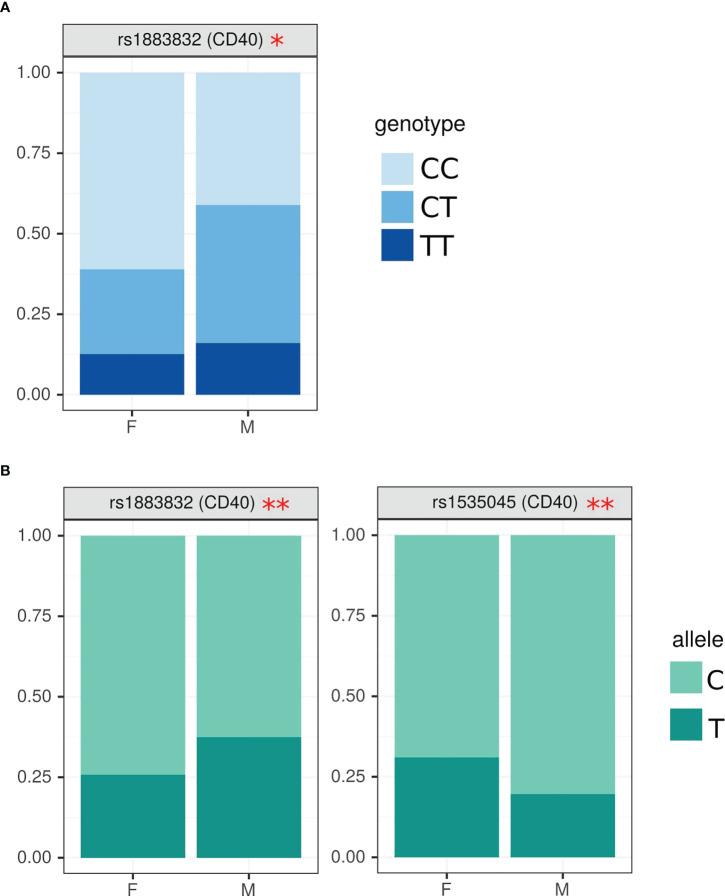
Analysis of SNPs genotype and allele frequency based on sex of MS patient. **(A)** – genotypes, **(B)** – alleles. SNPs analysis was done by AS-PCR. Data is presented as proportion of representation of genotype/allele. There were 151 MS patient samples used for the analysis. The analysis was done using Fisher’s exact test. ** - p<0.05, * - p<0.1.

When SNPs data was analyzed based on sex, CC genotype in rs1883832 of CD40 gene had a higher frequency in female MS (**Figure 2A**), while heterozygous CT genotype was often found in male MS (OR=2.40 (1.14; 5.09), p=0.05). We also found higher frequency of rs1883832^C^ and rs1535045^T^ in female as compared to male MS in CD40, (OR=1.72 (1.04; 2.85), p=0.04) and (OR=0.54 (0.31; 0.94), p=0.03), respectively (**Figure 2B**).

### Analysis of SNPs genotype and allele frequency in MS based on clinical presentation and presence of anti-herpesvirus antibodies

**Figure 3:**
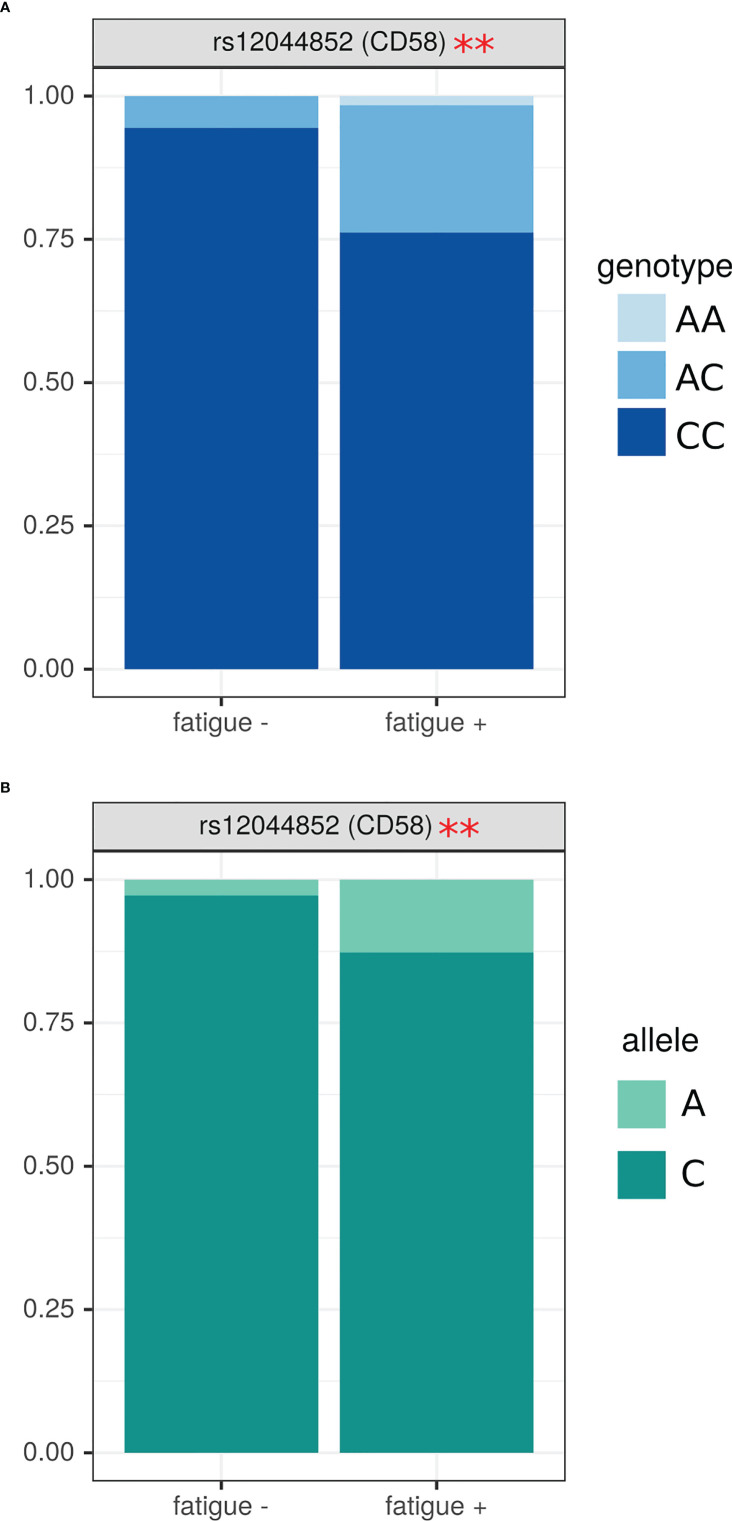
Analysis of SNPs genotype and allele frequency based on presence of fatigue in MS. **(A)** – genotypes, **(B)** – alleles. Data is presented as proportion of representation of genotype/allele. There were 151 MS patient samples used for the analysis. The analysis was done using Fisher’s exact test. ** - p<0.05.

Next, we analyzed SNPs genotype and allele frequency in patients with fatigue. For these analyses, we used data on 151 MS patients and 70 controls since age have a limited effect on the genotype. We have found that MS with fatigue had higher frequency of AC and AA genotypes in rs12044852 of *CD58* gene (OR=4.62 (1.17; 33.61), p=0.04) (**Figure 3A**). The higher frequency of allele A in patients with fatigue was also supported by the allele analysis in this SNP (**Figure 3B**).

**Figure 4:**
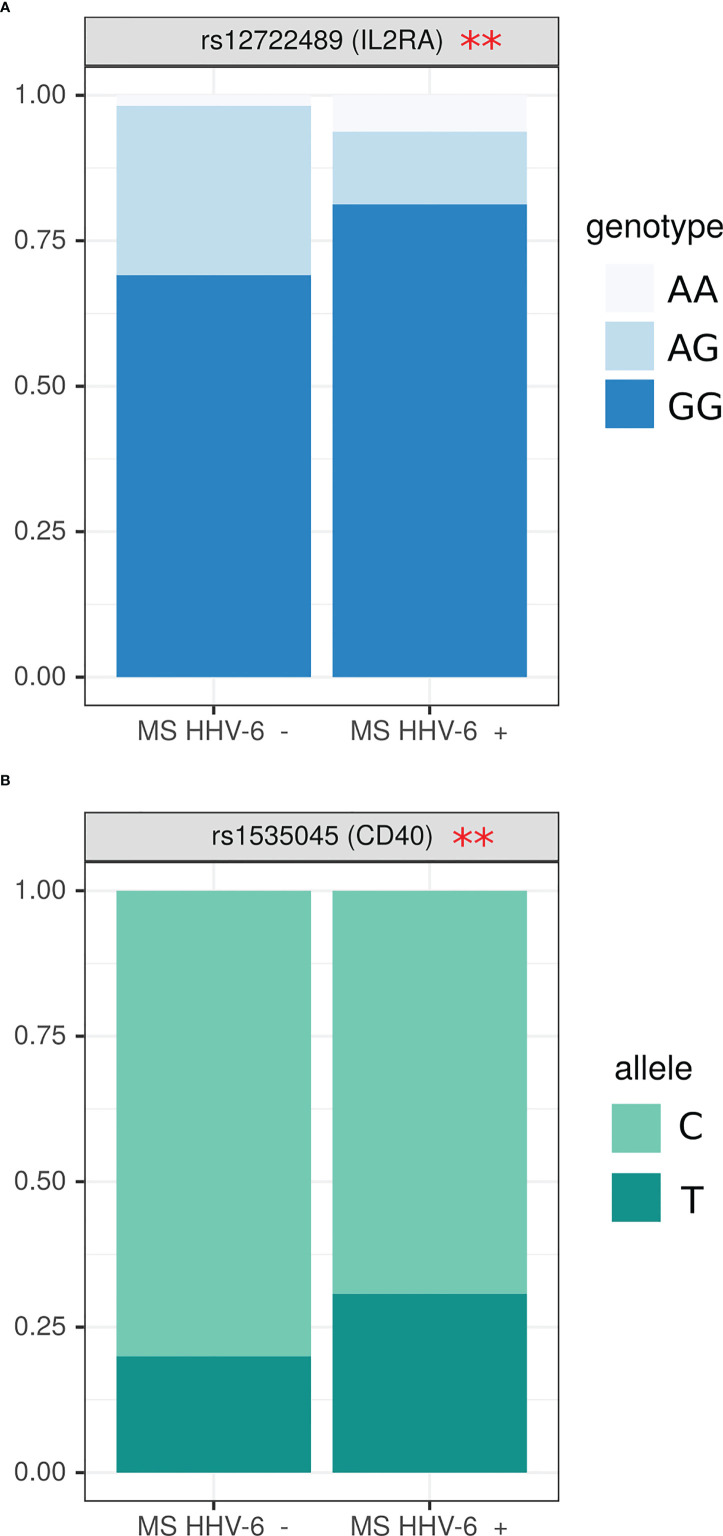
Analysis of SNPs genotype and allele frequency based on detection of circulating anti-HHV6 antibodies. **(A)** genotypes, **(B)** alleles. Data is presented as proportion of representation of genotype/allele. There were 151 MS patient samples used for the analysis. The analysis was done using Fisher’s exact test. ** - p<0.05.

Since our data demonstrated MS patients having higher HHV6 antibody prevalence as compared to controls, we sought to determine association between anti-herpesvirus antibodies and SNPs genotype. We have found that HHV6 antibody positive MS patients had more GG and less AG genotype in rs12722489 of IL2RA gene as compared to those without antibodies (**Figure 4A**) (OR=0.14 (0.005; 1.05), p=0.02). Also, the frequency of T allele in rs1535045 of *CD40* gene was higher in anti-HHV6 antibody positive patients as compared to those without anti-herpesvirus antibodies (OR=1.77 (1.02; 3.14), p=0.04) (**Figure 4B**).

**Figure 5:**
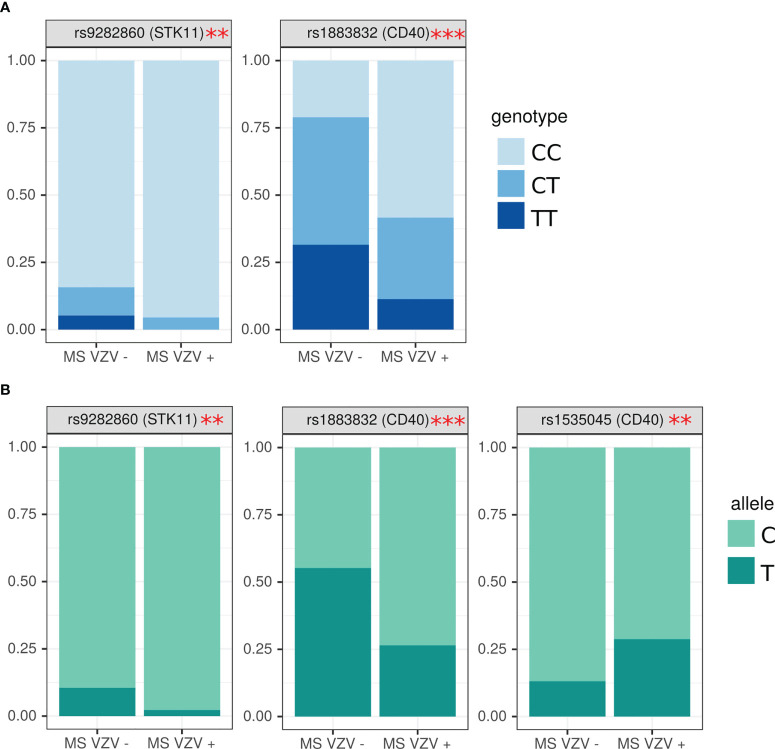
Analysis of SNPs genotype and allele frequency based on presence of circulating anti-VZV antibodies. **(A)** genotypes, **(B)** alleles. Data is presented as proportion of representation of genotype/allele. There were 151 MS patient samples used for the analysis. The analysis was done using Fisher’s exact test. *** - p<0.005, ** - p<0.05.

MS positive for anti-VZV antibodies were having higher frequency of CC genotype in rs9282860 (*STK11* gene) (OR=0.37, (0.07; 2.97) p=0.04) (**Figure 5A**). Interestingly, TT genotype in rs9282860 was absent in VZV seropositive. CC genotype was also more frequent in rs1883832 (CD40) of MS patients having anti-VZV antibodies as compared to those without (OR=0.24 (0.06; 0.79), p=0.003). Additionally, higher frequency of allele C in rs9282860 (*STK11* gene) and rs1883832 (*CD40* gene) was found in VZV seropositive as compared to seronegative MS (OR=0.19 (0.05, 0.84), p=0.03, OR=0.29 (0.14; 0.59) p=0.0006) (**Figure 5B**). T allele was more frequent in rs1535045 of *CD40* gene of anti-VZV antibody positive patients as compared to those without antibodies (OR=2.60 (1.05; 7.94) p=0.049).

## Discussion

Herpesvirus infections were linked to pathogenesis of MS in many studies. However, there is limited data on herpesvirus infection in the MS cohort from Russia, especially from the Volga Region of Russia. The population is a mix containing 66% Russians (Eastern Europeans), 13% Tatars and significant minorities of other groups such as the Bashkir (4.29%), Chuvash (4.26%), etc. Mixt ethnic ancestry is also frequent. This population diversity and composition differ substantially from that in Northern Europe, where high incidence and prevalence of MS are documented (59). MS is diagnosed in the Volga region of Russia, though the incidence (1.6-5.5 per 100,000 population) and prevalence (38-48 per 100,000 population) remain low (47) as compared to Northern Europe (59). Therefore, we sought to determine the prevalence of herpesvirus infection in MS and control cohorts from the Volga Region of Russia.

The current hypothesis of MS pathogenesis suggests multiple hits (60, 61), where one could be a herpesvirus infection, while the genetic risk factor could be the other. Therefore, we sought to analyze an association between herpesvirus infection and SNPs combination with MS diagnosis in the Volga Region of Russia cohort. Our knowledge of the MS associated SNPs in Russia is limited to several publications, including the patient’s cohort from Moscow and Novosibirsk (62–64). There is limited data on MS association with SNPs in the Volga Region of Russia cohort. Our data contribute to understanding the association between SNPs and MS from this region.

Our data confirms the potential link between herpesvirus infection and MS. We found significant association between HHV6 seropositivity and diagnosis of MS. This data corroborates previous observations where HHV6 infection was linked to MS (29, 65–67). Interestingly, EBV was also identified as a risk factor for MS (19, 24). In a comprehensive longitudinal study by Bjornevik et al., the association between EBV infection and MS diagnosis was thoroughly investigated (24) showing a 32 times higher risk of MS diagnosis after EBV seroconversion. Although our findings demonstrated a lack of connection between EBV and MS, we found more EBV seropositive samples (97.58%) in MS as compared to control (92.86%). This data supports a higher frequency of anti-EBV seropositivity in MS (68–70). A comprehensive analysis by Abrahamyan et al. revealed that most MS sera (93%) react with nuclear antigen (NA), while the remaining samples could react with viral capsid antigen (VCA) (22). The authors state that when reactivity to these viral antigens was analyzed, 100% EBV seropositivity was found in MS. We have used NA-based ELISA for anti-EBV antibody detection. Anti-NA antibodies are commonly used to establish EBV infection as they could be detected early (6-8 weeks after the exposure) and remain circulating for life (71). In contrast, anti-VCA antibodies indicate recent exposure as they could be found during the acute phase of the disease (72). Therefore, it appears that the detection of anti-NA antibodies would be a better indicator of the prior EBV infection. Still, anti-NA antibody negative results could not exclude the EBV infection, as some individuals’ serum could fail to react with anti-EBV NA (73).

Considering EBV as a risk factor for MS, it was interesting to find three EBV seronegative MS patients. Two patients were male, 41 and 42 years old. One patient was diagnosed with MS for one year and eight months. The patient did not receive treatment and had 6.5 and 9.47 scores for EDSS and MSSS, respectively. The other patient had a more extended history of MS diagnosis (22 years and three months). Unlike the first, this patient received disease-modifying treatment and had 4.0 and 1.64 scores for EDSS and MSSS, respectively. Interestingly, only this male patient was positive for HHV6 and VZV antibodies and VZV DNA. The third patient was a female with one year and ten months history of MS diagnosis. This patient did not receive disease-modifying treatment and had 6.5 and 1.77 scores for EDSS and MSSS, respectively. Interestingly, similar to the second male patient, antibodies to HHV6 and VZV as well as VZV DNA were detected in this female patient. These data support the role of herpesvirus infection in the pathogenesis of MS. It could be suggested to consider HHV6 and VZV as MS risk factors when an MS patient is negative for EBV antibodies and DNA.

Although herpesviruses were linked to MS diagnosis, the ubiquitous nature of these viruses and high seroprevalence could not entirely explain the MS global prevalence variations (74). Therefore, it could be suggested that additional factors, when combined with herpesvirus infection, contribute to the risk of MS. Genetic factors are often identified as markers of MS susceptibility, where multiple SNPs were shown to be associated with the likelihood of developing this disease. The study conducted by Zong-Li Xia et al., found correlation between genotypes in SNPs rs12722489 and rs2104286 (*IL2RA* gene) and MS diagnosis in the Hui and Han nationalities (75). The SNPs association rs6897932 was found in Jordanian MS patients (76). The rs9282860 in *STK11* gene was linked to a high likelihood of MS in women in the United States (77). Using meta-analysis, rs763361 in the *CD226* gene was shown to be linked to predisposition for having MS in Europe, Asia, Africa, and South America (78). In another study, SNP in the *CD6* gene rs17824933 was associated with MS in India, Europe, and the United states (79, 80). In cohorts in Greece, rs17445836 located in the long intergenic non-coding RNA near *IRF8* was identified as a risk factor for MS (81).

We have confirmed that the frequency of genotypes and alleles in selected genes differ between MS and controls. These data support the hypothesis of genetic risk factors of MS. We have found higher frequency of CC in rs12044852 and AA in rs2300747 genotypes in CD58 gene of MS. We also found that C allele in rs12044852 and A allele in rs2300747 in CD58 are risk factors for MS, while G allele in rs2300747 appears to be protective. These data corroborate de Jager, et al.’s findings, indicating protective role of rs2300747^G^ allele in MS (82). It appears that presence of this protective G allele is associated with higher expression of CD58 RNA (82, 83). Interestingly, the higher frequency of rs2300747^G^ allele in individuals with East Asian ancestry and associated increased level of *CD58* expression demonstrated by Purcell et al. (84) could contribute to the lower incidence and prevalence of MS shown in Asia (85). Our data support Torbati et al. data, indicating CC genotype in rs12044852 in CD58 as an MS risk factor (86). Our results also support the previous observation that the rs12044852^C^ allele is associated with MS in Caucasians (86), representing the majority in the Volga Region of Russia. Similar findings were reported by Hafler et al. in MS cohorts from the United States and Great Britain (87) as well as by Rubio et al. in Australian MS (88). In another study, d’Netto et al. presented data identifying rs12044852C as one of the non-HLA genetic risk factors (89). It could be suggested that the rs12044852^C^ allele had a diagnostic value as its combination DRB1*15 appears to better differentiate MS from controls than the HLA marker alone (90). The *CD58* gene encodes for a lymphocyte function-associated antigen 3 (LFA3), a glycosylated cell adhesion protein which is expressed on antigen presenting cells and function to enhance antigen presentation to T cells *via* interaction with CD2 (91, 92). This interaction was shown to be essential in antigen recognition and selection of primed T cells (93). Studies have shown that a protective G allele in rs2300747 genotype could cause an increased expression of CD58 which subsequently enhances function of CD4+CD25^high^ regulatory T cells *via* expression of transcription factor FoxP3 (82).

Another finding was a higher frequency of GG genotype in rs929230 of *CD6* in MS as compared to controls. The CD6 receptor is a transmembrane protein expressed on T-lymphocytes (94) and is involved in activation of lymphocytes by interacting with its ligand, the activated leukocyte cell adhesion molecule (ALCAM) [45]. Swaminathan et al. have demonstrated that rs17824933, located in the first intron of the gene, was more frequently found in MS within Spanish-Basque population (95). Similar association between this SNP and MS was demonstrated by de Jager et al. (82). We did not find the association between rs17824933 in CD6 and MS however, a higher frequency of allele G in rs929230 of the *CD6* gene in MS was presented. Interestingly, this SNP is also located in the first intron similar to rs17824933 (96), suggesting the role of this region of the gene in pathogenesis of MS.

When sex differences between MS patients we analyzed, we found higher frequency of C and T alleles in females as compared to males in CD40 rs1883832 and rs1535045, respectively. CD40 is a member of the TNF superfamily which is expressed on activated T and B cells (97, 98). Studies have shown that SNPs in *CD40* could be associated with MS depending on the ethnic and geographic location. In Australia, rs12044852 (*CD40* gene) was found more common in MS (99). However, in cohort from Iran, this mutation was not associated with MS risk [50]. Similar conclusions were made based on the study results of this SNP in patient populations from the USA and Europe (100). Our finding of sex distribution of alleles in rs1883832 and rs1535045 of *CD40* confirm the potential role of this gene in pathogenesis of MS (63, 101).

One interesting finding was a higher frequency of AC and AA genotypes in rs12044852 of the *CD58* genes in patients with fatigue symptoms. This is the first observation suggesting a connection between *CD58* gene SNPs and a symptom of MS. Fatigue is one most reported symptoms connected to an impaired quality of life (102, 103). Interestingly, altered CD58 expression was demonstrated in myalgic encephalitis, a disease characterizes by chronic fatigue (103).

An interesting observation made in this study was finding an association between frequency of alleles in *IL2RA* and *CD40* as well as seropositivity to HHV6 and VZV. Higher frequency of GG genotype in rs12722489 of *IL2RA* was found to be linked to HHV6 seropositivity. Interestingly, the G allele was also found linked to a high risk of MS (87, 104). The analysis of an association between this SNP and MS is shown to be the result of a linkage disequilibrium with another SNP, rs2104286 (Refining genetic associations in multiple sclerosis. 2008). Both SNPs were shown to influence IL-2RA function. The IL-2RA receptor coded by a gene containing the rs12722489^G^ allele had a higher affinity to estrogen receptor α (ERα) (105) Increased expression of IL-2RA was demonstrated in individuals having rs2104286 AA genotype (105), also known to be associated with MS. It was demonstrated that T helper cells from individuals carrying rs2104286 A SNP secrete more GM-CSF and have increased expression of IL-2RA. These data suggest that having a pathogenic allele in this SNP could support development of pathogenic GM-CSF expressing T helpers. IL-2RA is a component of the high affinity receptor for IL-2 (106). Therefore, the pathogenic effect of IL-2RA in MS could also be associated with IL-2 levels. Levels of IL-2 could be affected by HHV6 which suppress this cytokine production *via* expression of U54 protein (107). These data suggest that the role of IL-2RA in pathogenesis of MS could be more complex, and including regulatory cytokines, such as GM-CSF and IL-2, functions.

We also found an association between VZV sero-positivity and alleles C and T CD40 SNPs, rs1883832 and rs1535045, respectively. Interestingly, the same SNPs and alleles we found to be more frequent in females compared to male MS. CD40 SNP rs1883832 T>C was shown to be associated with increased MS risk (108). We found a higher frequency of CC genotype in females than in males in VZV infected MS. The CD40 rs1883832 was shown to have a substantial association with some form of autoimmunity. The association of rs1883832^C^ with Grave’s disease and rs1883832^T^ with MS was demonstrated in several studies (108–110). Interestingly, an association between rs1883832^T^ and MS was shown in several distinct Russian MS cohorts from Moscow and Siberia (63). In this view, finding more VZV infected MS females carrying CC CD40 rs1883832 genotype compared to males is intriguing. C allele in this SNP appears to be protective, since the risk of MS increases from 1.5 times in CT to 2.5 times in TT genotype individuals (111). It was shown that the C allele could enhance the efficiency of corresponding gene translation (108). Interestingly, the lower expression f CD40 was demonstrated in individuals with rs1883832^T^ compared to rs1883832^C^ CD40 SNPs (108, 112). Also, reduced expression of CD40 was demonstrated in MS (108). This reduced CD40 expression was shown in B lymphocytes, dendritic cells and monocytes in MS (108). Field et al. suggested that failure to develop tolerogenic mechanisms to explain the role of the low CD40 expression in MS pathogenesis (108). This CD40 tolerogenic immune response could be mediated by the stimulation of Tregs and the production of interleukin 10 (IL-10) (108). The higher frequency of rs1883832^C^ CD40 SNPs in VZV infected females MS as compared to males requires further investigation. Although we found no link between circulating VZV antibodies and MS diagnosis, which was due to high seroprevalence among controls and patients, confirming the ubiquitous nature of VZV infection. Still, there is much evidence suggesting VZV as a risk factor for MS [69-70]. Also, VZV could contribute to the progression of MS, as viral DNA was found in 100% of patients’ cerebrospinal fluid (CSF) samples during an exacerbation and in 31% of samples during remission [68]. Additionally more VZV DNA was found in CSF as compared to peripheral blood mononuclear cells in progressive MS [68].

**Figure 6:**
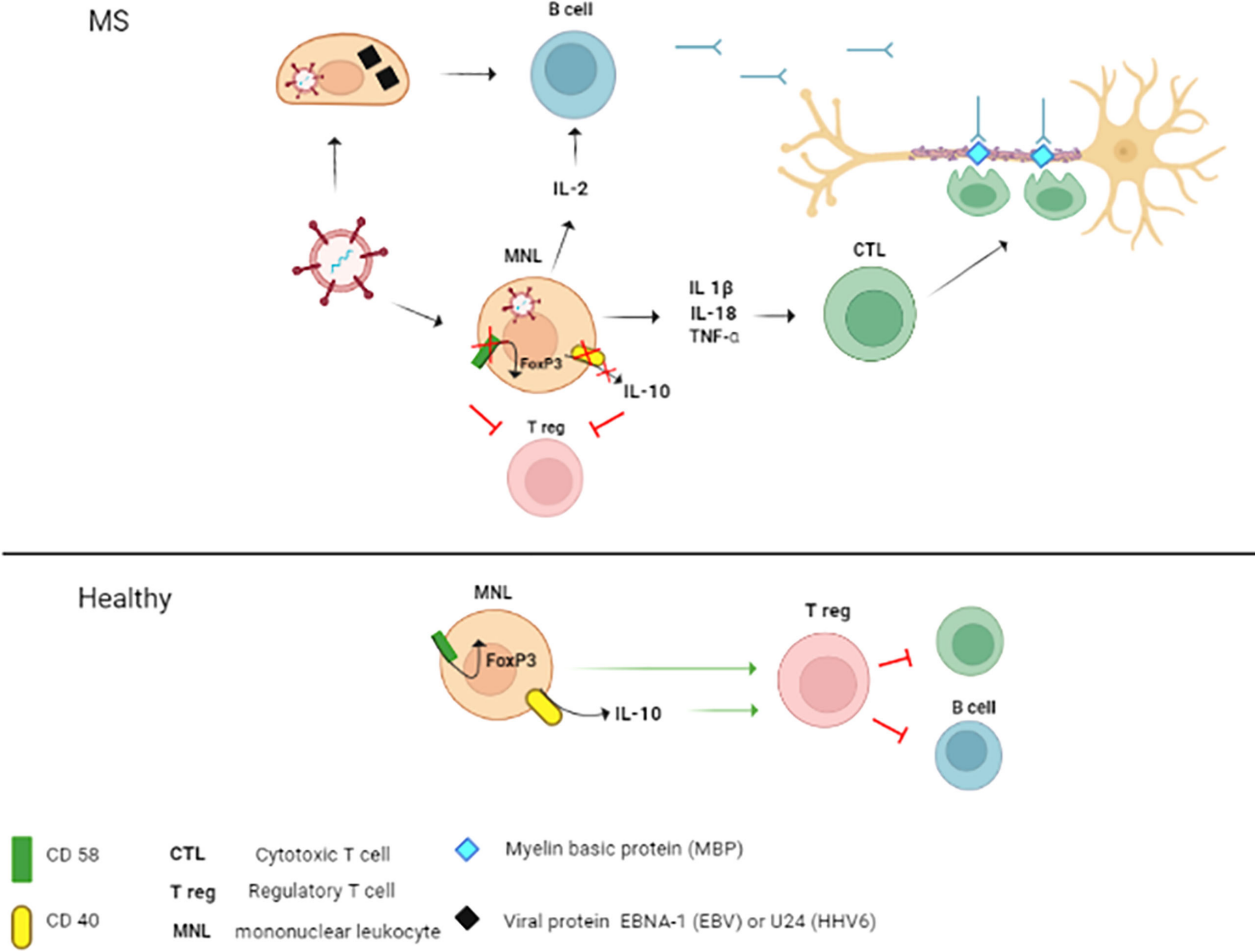
The mechanisms of the contribution of herpesvirus infection combined with CD58 and CD40 SNPs’ to MS pathogenesis. In MS, combined pathogenic effect of herpesviruses and SNPs in CD58 and CD40 could be explained by three potential mechanisms: 1. Molecular mimicry; 2. Activation and maintenance of inflammation; 3. Reduction of regulatory capacity of Tregs. 1. B cells primed with herpesvirus proteins (EBV nuclear antigen 1 (EBNA-1 and UL24) could produce antibody cross-reacting with myelin basic protein (MBP) epitopes (122,123). These antibodies could cross the blood-brain barrier (BBB) and target the axon myelin sheath. Also, cross-reactive cytotoxic T lymphocytes (CTL) could be generated and damage the axon myelin. 2. Herpesvirus infection could contribute to the pathogenesis of MS by activation of mononuclear leukocytes (MNL) and increasing the production of proinflammatory cytokines (IL-1β, IL-18 and TNF-α) (124–126). These cytokines could contribute to inflammation and promote activation and differentiation of pathogenic CTL (127,128).. Additionally, herpesviruses could activate autoreactive B cells directly and indirectly *via* the release of IL-2 by infected MNL (129,130). 3. The pathogenic role of SNPs in *CD58* and *CD40* could be associated with the reduction of Treg regulatory activity. This suggestion supports data demonstrating the association between the rs12044852^A^ allele in *CD58* and decreased expression of this surface marker (82). This low level of CD58 protein could consequently lead to the downregulation of FoxP3 (82), a regulatory element essential for the activation of Tregs (131). CD40 stimulates the production of IL-10 (132), a cytokine essential for Tregs signaling (133). Also, CD40 was shown to augment the tolerogenic effect of IL-10 (134). Therefore, rs1883832^T^ associated reduction of CD40 expression could impact the Tregs function in MS. Also, suppression of Tregs could be caused by lower expression of CD40 in individuals with 1883832^T^ allele (101,135). In healthy individuals, expression of CD40 and CD58 remains unaffected. Also, Tregs signaling would be supported by release of IL-10 and expression of FoxP3.

In conclusion, we have found an association between HHV6 sero-positivity and MS diagnosis. This data contributes to our understanding the role of herpesvirus infection as risk factor of MS. Two viruses, EBV and HHV6, are frequently associated with MS (24, 67, 113, 114). Also, childhood herpesvirus infection increases the risk of MS diagnosis (115). Therefore, detection of anti-herpesvirus antibodies should be considered as a potential risk of MS diagnosis later. Also, studies demonstrated the association between HHV6 reactivation and MS relapse (116, 117). Therefore, the therapeutic potential of anti-herpesvirus drugs was evaluated in some studies showing limited efficacy (118, 119). However, these therapeutics could be used to control herpesvirus reactivation caused by alemtuzumab (120), a drug also used for MS treatment (121). We also confirmed higher frequency of A, C and G alleles in rs2300747 and rs12044852 of CD58 and rs929230 in CD6 in MS as compared to controls. We found higher frequency of C and T alleles in female as compared to male in CD40 rs1883832 and rs1535045, respectively. The herpesvirus infection and pathogenic SNPs combined mechanisms of MS pathogenesis are summarized in **Figure 6**. Fatigue symptoms were the only clinical symptoms linked to AC and AA genotype in rs12044852 of *CD58* gene. The interesting observation, supporting the role of HHV6 in MS pathogenesis, was finding a higher frequency of GG genotype in rs12722489 of *IL2RA* and T allele in rs1535045 of *CD40* genes in patient having anti-HHV6 antibodies as well as a link between having anti-VZV antibodies in MS and CC genotype in rs1883832 of *CD40* gene.

## Data availability statement

The original contributions presented in the study are included in the article/**Supplementary Material**. Further inquiries can be directed to the corresponding author.

## Ethics statement

The studies involving human participants were reviewed and approved by Biomedicine Ethic Expert Committee of Republican Clinical Neurological Center, Republic of Tatarstan, Russian Federation (N: 218, 15.11.2012). Written informed consent for participation was not required for this study in accordance with the national legislation and the institutional requirements.

## Author contributions

AZ, AM – investigation. MM - visualization. SK – conceptualization. VL, YD, EM, SK - writing, review and editing. All authors contributed to the article and approved the submitted version.

## Funding

This work was supported by the Kazan Federal University Strategic Academic Leadership Program (PRIORITY-2030).

## Conflict of interest

The authors declare that the research was conducted in the absence of any commercial or financial relationships that could be construed as a potential conflict of interest.

## Publisher’s note

All claims expressed in this article are solely those of the authors and do not necessarily represent those of their affiliated organizations, or those of the publisher, the editors and the reviewers. Any product that may be evaluated in this article, or claim that may be made by its manufacturer, is not guaranteed or endorsed by the publisher.
