# Supplementary material for: Analysis of herpesvirus infection and genome single nucleotide polymorphism risk factors in multiple sclerosis, Volga federal district, Russia
Source: Front Immunol. 2022 Nov 14;13:1010605. doi: 10.3389/fimmu.2022.1010605 (PMC9703080; doi:10.3389/fimmu.2022.1010605)
Supplement: Supplementary file 1 [file DataSheet_1.docx]

Supplemental Figure 1. The results of AS-PCR products separation by gel electrophoresis for rs1883832:

1-22 – samples; C+ - positive control; C- - negative control; M – DNA Ladder

Supplemental Table 1A. Analysis of herpesvirus antibodies and DNA in female MS and control

|  | | CTRL female (n (%)) | | MS female (n (%)) | | Fisher's Exact Test |
| --- | --- | --- | --- | --- | --- | --- |
| Method | Herpesvirus | + | - | + | - | p value |
| ELISA | CMV | 38 (79.17%) | 10 (20.83%) | 61 (81.33%) | 14 (18.67%) | 0.82 |
|  | HHV6 | 23 (47.92%) | 25 (52.08%) | 46 (61.33%) | 29 (38.67%) | 0.19 |
|  | EBV | 46 (95.83%) | 2 (4.17%) | 74 (98.67%) | 1 (1.33%) | 0.56 |
|  | VZV | 37 (77.08%) | 11 (22.92%) | 67 (89.33%) | 8 (10.67%) | 0.08 |
| PCR | CMV | 0 (0.00%) | 40 (100.00%) | 0 (0.00%) | 75 (100.00%) | 1 |
|  | HHV6 | 1 (2.50%) | 39 (97.50%) | 1 (1.33%) | 74 (98.67%) | 1 |
|  | EBV | 1 (2.50%) | 39 (97.50%) | 0 (0.00%) | 75 (100.00%) | 1 |
|  | VZV | 11 (27.50%) | 29 (72.50%) | 18 (24.00%) | 57 (76.00%) | 0.82 |

Supplemental Table 1b. Analysis of herpesvirus antibodies and DNA in male MS and control

|  | | Control male (n (%)) | | MS male (n (%)) | | Fisher's Exact Test |
| --- | --- | --- | --- | --- | --- | --- |
| Method | Herpesvirus | + | - | + | - | p value |
| ELISA | CMV | 20 (90.91%) | 2 (9.09%) | 44 (89.80%) | 5 (10.20%) | 1 |
|  | HHV6 | 9 (40.91%) | 13 (59.09%) | 33 (67.35%) | 16 (32.65%) | 0.07 |
|  | EBV | 19 (86.36%) | 3 (13.64%) | 47 (95.92%) | 2 (4.08%) | 0.17 |
|  | VZV | 19 (86.36%) | 3 (13.64%) | 42 (85.71%) | 7 (14.29%) | 1 |
| PCR | CMV | 0 (0.00%) | 19 (100.00%) | 0 (0.00%) | 49 (100.00%) | 1 |
|  | HHV6 | 0 (0.00%) | 19 (100.00%) | 0 (0.00%) | 49 (100.00%) | 1 |
|  | EBV | 0 (0.00%) | 19 (100.00%) | 1 (2.04%) | 48 (97.96%) | 1 |
|  | VZV | 2 (10.53%) | 17 (89.47%) | 11 (22.45%) | 38 (77.55%) | 0.33 |

N – number of patients; % - percent of patients from total number in the group.

Supplemental Table 2A. Analysis of herpesvirus antibodies and DNA in younger (≤35 years old) MS and control

|  | | Control ≤35 years old (n (%)) | | MS ≤35 years old (n (%)) | | Fisher's Exact Test |
| --- | --- | --- | --- | --- | --- | --- |
| Method | Herpesvirus | + | - | + | - | p value |
| ELISA | CMV | 35 (81.40%) | 8 (18.60%) | 58 (90.63%) | 6 (9.38%) | 0.24 |
|  | HHV6 | 21 (48.84%) | 22 (51.16%) | 40 (62.50%) | 24 (37.50%) | 0.17 |
|  | EBV | 42 (97.67%) | 1 (2.33%) | 63 (98.44%) | 1 (1.56%) | 1 |
|  | VZV | 34 (79.07%) | 9 (20.93%) | 53 (82.81%) | 11 (17.19%) | 0.62 |
| PCR | CMV | 0 (0.00%) | 36 (100.00%) | 0 (0.00%) | 61 (100.00%) | 1 |
|  | HHV6 | 0 (0.00%) | 36 (100.00%) | 0 (0.00%) | 61 (100.00%) | 1 |
|  | EBV | 0 (0.00%) | 36 (100.00%) | 1 (1.64%) | 60 (98.36%) | 1 |
|  | VZV | 3 (8.33%) | 33 (91.67%) | 18 (29.03%) | 44 (70.97%) | **0.02** |

Supplemental Table 2B. Analysis of herpesvirus antibodies and DNA in older (>35 years old) MS and control

|  | | Control >35 years old | | MS >35 years old | | Fisher's Exact Test |
| --- | --- | --- | --- | --- | --- | --- |
| Method | Herpesvirus | + | - | + | - | p value |
| ELISA | CMV | 23 (85.19%) | 4 (14.81%) | 47 (78.33%) | 13 (21.67%) | 0.57 |
|  | HHV6 | 11 (40.74%) | 16 (59.26%) | 39 (65.00%) | 21 (35.00%) | **0.04** |
|  | EBV | 23 (85.19%) | 4 (14.81%) | 58 (96.67%) | 2 (3.33%) | 0.073 |
|  | VZV | 22 (81.48%) | 5 (18.52%) | 56 (93.33%) | 4 (6.67%) | 0.13 |
| PCR | CMV | 0 (0.00%) | 23 (100.00%) | 0 (0.00%) | 60 (100.00%) | 1 |
|  | HHV6 | 1 (4.35%) | 22 (95.65%) | 1 (1.67%) | 59 (98.33%) | 1 |
|  | EBV | 1 (4.35%) | 22 (95.65%) | 0 (0.00%) | 60 (100.00%) | 1 |
|  | VZV | 10 (43.48%) | 13 (56.52%) | 11 (18.33%) | 49 (81.67%) | **0.03** |

Supplemental Table 3. Analysis of EDSS and MSSS in MS patients and detection of herpesvirus antibodies and DNA

|  | | EDSS | | MSSS | | Kruskal-Wallis rank sum test, p value | |
| --- | --- | --- | --- | --- | --- | --- | --- |
| Method | Herpesvirus | + (n; mean±SD) | - (n; mean±SD) | + (n; mean±SD) | - (n; mean±SD) | EDSS | MSSS |
| ELISA | CMV | 125; 2.85±1.55 | 26; 3.00±1.83 | 114; 4.42±2.38 | 26; 3.88±2.99 | 0.86 | 0.15 |
|  | HHV6 | 96; 2.71±1.38 | 55; 3.15±1.90 | 87; 4.20±2.43 | 53; 4.52±2.62 | 0.37 | 0.56 |
|  | EBV | 146; 2.85±1.60 | 5; 3.50±1.84 | 135; 4.33±2.46 | 4; 4.16±3.87 | 0.31 | 0.60 |
|  | VZV | 132; 2.86±1.54 | 19; 3.00±2.04 | 121; 4.19±2.46 | 19; 5.17±2.68 | 0.80 | 0.10 |
| PCR | CMV | 0; NA | 151; 2.87±1.60 | 0; NA | 140; 4.32±2.50 | NA | NA |
|  | HHV6 | 1; 4±NA | 150; 2.87±1.60 | 1; 5.28±NA | 139; 4.32±2.51 | 0.40 | 0.61 |
|  | EBV | 1; 3±NA | 150; 2.87±1.61 | 0; NA | 140; 4.32±2.50 | 0.79 | NA |
|  | VZV | 37; 2.73±1.62 | 114; 2.92±1.60 | 32; 4.18±2.48 | 108; 4.36±2.52 | 0.38 | 0.69 |

+ (n; mean±SD) – herpesvirus ELISA/PCR positive;

- (n; mean±SD) – herpesvirus ELISA/PCR negative.

Supplemental Table 4. Analysis of presence of treatment in MS patients and detection of herpesvirus antibodies and DNA

|  | | MS treatment (+) | | MS treatment (-) | | Fisher's Exact Test |
| --- | --- | --- | --- | --- | --- | --- |
| Method | Herpesvirus | + | - | + | - | p value |
| ELISA | CMV | 61 (88.41%) | 8 (11.59%) | 44 (80.00%) | 11 (20.00%) | 0.22 |
|  | HHV6 | 47 (68.12%) | 22 (31.88%) | 32 (58.18%) | 23 (41.82%) | 0.27 |
|  | EBV | 68 (98.55%) | 1 (1.45%) | 53 (96.36%) | 2 (3.64%) | 0.58 |
|  | VZV | 61 (88.41%) | 8 (11.59%) | 48 (87.27%) | 7 (12.73%) | 1 |
| PCR | CMV | 0 (0.00%) | 69 (100.00%) | 0 (0.00%) | 55 (100.00%) | 1 |
|  | HHV6 | 1 (1.45%) | 68 (98.55%) | 0 (0.00%) | 55 (100.00%) | 1 |
|  | EBV | 1 (1.45%) | 68 (98.55%) | 0 (0.00%) | 55 (100.00%) | 1 |
|  | VZV | 14 (20.29%) | 55 (79.71%) | 15 (27.27%) | 40 (72.73%) | 0.40 |

MS treatment (+) – having immunomodulating treatment;

MS treatment (-) - not having immunomodulating treatment.
